# Feasibility of Left Atrial Appendage Closure in Atrial Fibrillation Patients with a History of Intracranial Bleeding: A Systematic Review of Observational Studies

**DOI:** 10.1155/2020/1575839

**Published:** 2020-11-06

**Authors:** Muhammad Ajmal, Qurat Ul Ain Riaz Sipra, Cristina Pecci, Nusrum Iqbal, Sulaiman Rathore

**Affiliations:** ^1^Cardiology Fellow at University of Arizona, Tucson, AZ, USA; ^2^Internal Medicine Resident at University of Arizona, Tucson, AZ, USA; ^3^Cardiology Fellow at University of Arizona, Phoenix, AZ, USA; ^4^Hospital Medicine at St.Mary's Hospital, Tucson, AZ, USA; ^5^Interventional and Structural Cardiologist at Northwest Hospital, Tucson, AZ, USA

## Abstract

**Background:**

Left atrial appendage occlusion (LAAO) is performed in patients with nonvalvular atrial fibrillation to reduce the risk of ischemic stroke. The patients with a history of intracranial hemorrhage were excluded from the pioneer randomized controlled trials. The purpose of this systemic review was to evaluate the data from observational studies reporting the efficacy and safety of LAAO in patients with a history of intracranial hemorrhage.

**Methods:**

Ovid MEDLINE, Embase, Cochrane Central Register of Controlled Trials, Web of Science Core Collection, Scopus, Global Index Medicus, and ClinicalTrials.gov data sources were utilized for data collection.

**Results:**

A total of 12 studies met the inclusion criteria that included seven retrospective observational and five prospective observational studies. A total of seven hundred and twenty-seven patients with a history of intracranial hemorrhage underwent percutaneous left atrial appendage occlusion. There were 11 events of recurrent intracranial hemorrhage, 12 ischemic strokes, 4 transient ischemic attacks, and 26 all-cause deaths. The duration of follow-up varied from 3 months to 3.6 years in the included studies.

**Conclusion:**

Left atrial appendage occlusion can potentially be an effective and relatively safe treatment option to reduce the risk of ischemic stroke in selected patients with nonvalvular atrial fibrillation patients and history of intracranial hemorrhage. Future prospective randomized trials are needed to validate this approach.

## 1. Introduction

Atrial fibrillation is associated with a high arterial thromboembolic risk, leading to increased morbidity and mortality. The use of anticoagulants (warfarin or direct oral anticoagulants) effectively reduces the risk of thrombosis [1, 2]. However, the side effect profile of oral anticoagulants restricts their use in patients who have a history of major bleeding or if they are at increased risk of bleeding.

Approximately, 90% of thrombus in atrial fibrillation are formed in the left atrial appendage. Therefore, mechanical left atrial appendage occlusion (LAAO) is a novel therapeutic modality used to reduce the risk of atrial thromboembolism in patients with nonvalvular atrial fibrillation (AF) and increased risk of bleeding [3, 4]. The two pivotal trials, PROTECT AF (Watchman Left Atrial Appendage System for Embolic Protection in Patients with Atrial Fibrillation) and PREVAIL (Evaluation of the Watchman LAA Closure Device in Patients with Atrial Fibrillation Versus Long Term Warfarin Therapy), evaluated the effectiveness of LAAO with Watchman device (Boston Scientific, St. Paul, Minnesota) compared with warfarin [3, 5]. The results of these two trials demonstrated that LAAO is noninferior to warfarin in preventing ischemic stroke and is superior in preventing cardiovascular and all-cause mortality. Subsequently, the Watchman device was approved by the United States Food and Drug Administration (FDA) in 2015 [5]. In 2019, American Heart Association/American College of Cardiology/Heart Rhythm Society recommended LAAO for patients who are at increased risk for thromboembolism and have contraindications to anticoagulation [6].

In PROTECT AF and PREVAIL trials, individuals with a history of an intracranial hemorrhage were excluded due to the perceived risk of recurrent intracranial hemorrhage perioperatively [5]. However, in real-world, LAAO is being used in AF patients with a history of intracranial hemorrhage and there are emerging data from observational studies and registries that this modality might be safe to use [7].

Despite the increasing use of LAAO procedure in AF patients with a history of intracranial hemorrhage, the data regarding efficacy and safety are limited. Therefore, we conducted a systematic review of literature to evaluate the efficacy and safety of mechanical left atrial appendage occlusion in nonvalvular atrial fibrillation patients with a history of intracranial hemorrhage.

## 2. Methods

### 2.1. Data Sources and Searches

Protocol for the review was developed in February 2020. We searched Ovid MEDLINE, Embase, Cochrane Central Register of Controlled Trials, Web of Science Core Collection, Scopus, Global Index Medicus, and ClinicalTrials.gov through April 3^rd^, 2020, without study design or language restriction. The study protocol is provided in Supplementary Material 1 and search categories are provided in Supplementary Material 2.

### 2.2. Study Selection

**Figure 1:**
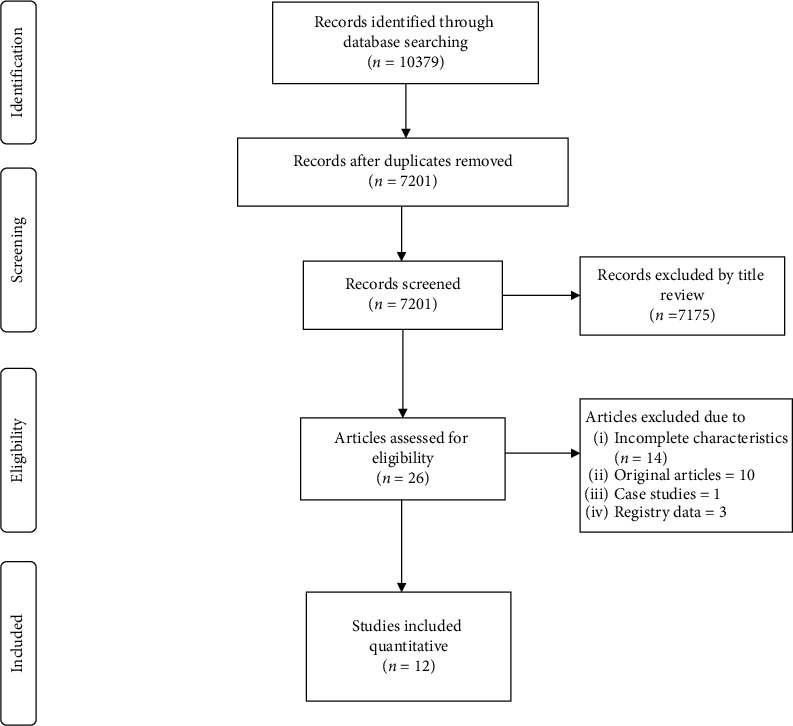
PRISMA LAAO in intracranial hemorrhage.

This systematic review is reported according to the Preferred Reporting Items for Systematic Reviews and Meta-Analyses (PRISMA) statement [8]. Articles were screened and selected by 2 independent reviewers using 3-step approach. First, all the articles mentioning left atrial appendage closure were screened, and there were a total of 10379 articles; after removing duplicates, the remaining articles were 7201. Then out of these, articles reporting left atrial appendage closure in intracranial hemorrhage and/or high-risk patients were reviewed for relevance, and 26 articles were assessed for eligibility. After that, articles with incomplete characteristics or the ones not reporting separate bleeding or thromboembolic risk scores for the intracranial hemorrhage cohort were removed, and the final 12 observational studies were extracted for final analysis. These steps are shown in the PRISMA file (Figure 1).

### 2.3. Outcomes

The outcomes of interest included device-related thrombus (DRT), ischemic stroke/TIA, recurrence of intracranial hemorrhage, and all-cause mortality.

### 2.4. Data Extraction and Quality Assessment

Two reviewers independently extracted data on baseline characteristics, type of intracranial hemorrhage, duration between intracranial hemorrhage and device implantation, type of devices used, antithrombotic and their duration after device implant, follow-up and outcomes of device-related thrombus, ischemic stroke, recurrent intracranial hemorrhage, and mortality. Quality of the studies was assessed by two reviewers using National Heart, Lung and Blood Institute (NIH) Quality Assessment Tool for Observational Cohort and Cross-Sectional Studies [9].

### 2.5. Data Synthesis and Analysis

Data were summarized using descriptive statistics, with means, medians, and ranges for continuous variables and frequencies and percentages for dichotomous variables.

## 3. Results

### 3.1. Study Selection

We identified 7201 articles, and out of these, 12 observational studies (*n* = 727) met the inclusion criteria. There were seven retrospective and five prospective studies. The study characteristics are summarized in Table 1.

### 3.2. Patient Characteristics

A total of 727 patients underwent LAAO, in which 65.1% were males and 34.9% were females. Mean age was 74.1 ± 2.2. CHA2DS2-VASc risk score and HAS-BLED risk scores were reported by mean and median in different studies as shown in Table 1. The type of intracranial hemorrhage was not reported in 3 studies that contributed 358 patients from a total cohort of 727 patients. Out of 365 patients, 71% had intracranial hemorrhage, 19% had subdural hemorrhage, and 7.5% had subarachnoid hemorrhage, while 2.5% had microhemorrhages, ocular bleeding, or other bleedings. The data for four patients were missing. The duration between intracranial hemorrhage and LAAO procedure varied among studies, but the average duration was about two months, as shown in Table 1. All three major devices were used in all the procedures which included Watchman, Amplatzer Cardiac Plug, and Amplatzer Amulet.

### 3.3. Outcomes

Primary outcomes were device-related thrombus (DRT), ischemic stroke/TIA, recurrence of intracranial hemorrhage, and mortality. The results of primary outcomes are summarized in Table 2. DRT  was not reported in 3 studies which included 264 patients, and in the remaining 463 patients, there were 6 DRT (1.3%). The ischemic stroke and TIA were reported in all studies which were 12 (1.6%) and 4 (0.5%), respectively. The recurrence of intracranial hemorrhage was reported in all 12 studies with a total of 11 events (1.5%). The overall mortality rate was 3.6% (26/727). Twenty-two deaths were reported in one study of 104 patients with a median follow-up of 3.6 years by Pouru et al. (Table 2). This cohort included a total of 75% arterial thromboembolic events, 64% recurrent intracranial hemorrhage, and 85% deaths reported in our study.

There were limited data on the day of occurrence of the event and which antithrombotic regimen patients were on at the time of primary outcomes. Follow-ups were reported in absolute days or months, mean with standard deviation (SD), or median with interquartile range (IQR) in different studies, as shown in Table 2. The duration of follow-up varied from 3 months to 3.6 years depending upon the study.

### 3.4. Antithrombotic Regimen and Duration

Antithrombotic regimen after LAAO was reported in 674 patients, and the choice of regimen and duration varied among included studies. The shortest duration of anticoagulation with warfarin, DOACs, and LMWH was reported ≤14 days in 17/674 (2.5%) patients and ≤1 month in 31/674 (4.6%) patients. Anticoagulation for first 6 weeks was used in 106/674 (15.7%) of patients which included warfarin (50/106 (47%)), direct oral anticoagulants (54/106 (51%)), and low molecular weight heparin (2/106 (2%)). Out of these 106 patients, 43 (40.5%) were also on aspirin along with oral anticoagulation. 81/106 (76.4%) patients were continued on dual antiplatelet (aspirin and clopidogrel) for the next 4.5 months and then low-dose aspirin lifelong. Only one study reported the use of warfarin for 3 months in a patient.

Dual antiplatelet therapy (DAPT) with aspirin and clopidogrel was used in 134/674 (19.9%) of patients after LAAO. DAPT  for first 6 weeks was used in 50/674 (7.4%) of patients, for 1 month in 12/674 (1.8%), for 1.5 months in 38/674 (5.6%), for 3 months in 32/674 (4.7%), and for 6 months in 2/674 (0.3%). After DAPT, lifelong aspirin was reported in 180 patients.

31/674 (4.6%) patients were not on any antithrombotic after left atrial appendage occlusion. The detail of the antithrombotic regimen is shown in Table 3.

### 3.5. Quality Assessment

The quality of the studies was low-to-moderate based on the NIH quality assessment tool, which is reported in Supplementary Material 3. Titles of all the articles were relevant to the study question, and baseline characteristics were well described in all the studies. 9 out of 12 studies reported the type of intracranial hemorrhage, and most of the studies reported duration between intracranial hemorrhage and device implant. Antithrombotic regimen after the procedure and device-related thrombus (DRT) were reported in most of the studies. Primary outcomes of recurrence of intracranial hemorrhage, transient ischemic attack (TIA)/stroke, and mortality were reported in all the studies.

## 4. Discussion

The findings from our study suggest that left atrial appendage occlusion can offer an effective treatment option with a satisfactory safety profile to reduce the risk of ischemic stroke in selected patients with nonvalvular atrial fibrillation and history of intracranial hemorrhage. There are several challenges in establishing the efficacy and safety of available treatment options for this high-risk cohort of atrial fibrillation. Based on the average CHADSD2-VASc and HAS-BLED risk scores, the inherent predisposition to a higher risk for arterial thromboembolism from atrial fibrillation and major bleeding from antithrombotic therapies are the most important considerations. In addition, this cohort mostly represents the geriatric population who are more likely to be frail and high risk for falls and injuries and, subsequently, higher risk of adverse intracranial hemorrhagic events [10].

The results of our study are supporting the emerging evidence indicating the safety and efficacy of LAAO with the perioperative short-term use of anticoagulation in patients with contraindications to anticoagulation including a history of intracranial hemorrhage. Barakat et al. reported no recurrence of spontaneous bleeding in 20 consecutive patients with contraindications to anticoagulation including 7 of whom had a history of intracranial hemorrhage [11]. Antithrombotic regimen included anticoagulation for 45 days, followed by 4.5 months of dual antiplatelet with aspirin and clopidogrel and then lifelong aspirin. J.R. Lopez-Minguez studied 598 patients who underwent LAA occlusion at 13 tertiary care centers across the Iberian Peninsula, which included 160 patients with previous intracranial hemorrhage. At a mean follow-up of 22.9 months, there was a 0.8% recurrence of intracranial hemorrhage and, at a follow-up of >24 months, recurrence of intracranial hemorrhage was 0.4% and the expected recurrence of bleeding based on the HAS-BLED score was 0.9% and 0.8%, respectively [12]. The antithrombotic regimen consisted of 600 mg loading of clopidogrel with aspirin 300 mg on the first day, followed by 100 mg aspirin daily for at least 6–12 months along with clopidogrel 75 mg daily for 3–6 months.

ASAP (ASA Plavix Feasibility Study with Watchman Left Atrial Appendage Closure Technology) is a nonrandomized, prospective study, which evaluated the safety and efficacy of LAAO in high-risk population. This cohort of 150 patients had contraindications to anticoagulation and was at high risk for ischemic stroke. These patients underwent LAAO and received dual antiplatelets with aspirin and clopidogrel for 6 months without receiving warfarin and then lifelong aspirin [13]. The recurrence of intracranial hemorrhage and thromboembolism reported in the high-risk population of ASAP study, although the design and patients' characteristics were different, was 0.7% and 2.6%, while our systemic review containing 727 patients reports recurrent intracranial hemorrhage of 1.5% and thromboembolism (TIA and stroke) in 2.2% of patients. The review of 4 prospective clinical trials reporting device-related thrombus (DRT) in the clinical trials of left atrial appendage occlusion including 1739 patients by Dukkipati et al. found that DRT was 3.7% compared to 1.3% in 464 patients in our study [14].

After comparing the results of our study to other studies of the high-risk population (population at high risk for stroke and bleeding), we propose that left atrial appendage occlusion in patients with nonvalvular atrial fibrillation and a history of intracranial hemorrhage is safe and effective. We also propose that the timing between the intracranial bleeding event and LAAO should be individualized and decided by a multidisciplinary approach. Regarding the antithrombotic regimen, we suggest that strategy of anticoagulation for 6 weeks after LAAO followed by 4.5 months of DAPT and then lifelong aspirin or DAPT (aspirin and clopidogrel) without the use of anticoagulation for 6 months after LAAO followed by lifelong aspirin can be considered.

This review provides the most up to date evidence of left atrial appendage occlusion in patients with nonvalvular atrial fibrillation and a history of intracranial hemorrhage. There are several limitations. The conclusions are primarily drawn from the observational studies with no comparator group, which poses a potential risk of confounding and bias. There is a potential for a higher likelihood of invalidity because of the lack of standardized approach for the assessment and treatment, and the potential for loss of follow-up might have affected the interpretation of the results. There was also a lack of generalized approach for the antithrombotic regimen postprocedure compared to the pioneer trials, and the timing of the procedure after intracranial hemorrhage was also not uniform and we cannot generalize one approach to all the patients. The included studies did not report safety and efficacy comparison among different left atrial appendage devices, and we cannot conclude the superiority of any device. Pouru et al. (Table 2) reported more safety events compared to the other studies, although we can speculate that it might be related to the longer duration of follow-up with a mean follow-up of 3.6 years compared to the other studies but the author is unable to conclude any significant reason for the higher safety events, as the baseline characteristics in this study were comparable to other studies. Finally, the duration of follow-up in the included studies was relatively short, and longer follow-up might lead to different outcomes.

In summary, the findings from our study indicate that LAAO may be considered in selected patients with nonvalvular atrial fibrillation and a history of intracranial hemorrhage. Although the patient population with a history of intracranial hemorrhage is at high risk for both recurrent intracranial hemorrhage and systemic arterial thromboembolism, multidisciplinary approach should be opted for the safety of LAAO and short-term anticoagulation in these cases. The selected patients after shared decision-making may undergo this procedure successfully, and the anticoagulation regimen and duration may be tailored to the individual patients. Future prospective randomized clinical trials are needed to validate this approach.

## Figures and Tables

**Table 1:** Baseline characteristics of all studies

Author/year	Study type	No. of pts	Male, no. (%)	Female, no. (%)	Age, mean ± SD	CHA2DS2-VASc score, mean ± SD; median ± IQR	HAS-bled score, mean ± SD; median ± IQR	Type of intracranial hemorrhage	Duration between device implant and intracranial hemorrhage
Ajmal/2020	Retrospective observation	16	9 (56.2%)	7 (43.8%)	74.6 ± 5.8	Median 4.5; IQR 3	Median 4; IQR 1	7 IPH 7 SDH 2 SAH	More than 2 months
Fayos-Vidal/2020	Retrospective observation	9	7 (77.7%)	2 (22.3%)	72.7 ± 8.2	Median 4; IQR 2.5	Median 3; IQR 0	8 IPH 1 SDH	Less than 1 months in 5 More than 1 months in 4
Pouru/2020	Prospective registry	104	73 (70.1%)	31 (29.9%)	73 ± 7	Mean ± SD: 4.7 ± 1.4	Mean ± SD: 3.3 ± 0.9	69 IPH 21 SDH 11 SAH 2 not reported	Median: 7 Months
Hucker/2019	Retrospective observation	63	37 (58.7%)	26 (41.3%)	75.3 ± 6.0	Mean ± SD: 4.9 ± 1.7	Mean ± SD: 3.5 ± 1.1	36 IPH 18 SDH 6 SAH 3 uncertain	Median: 212 days; IQR: 78–548 days
Hutt/2019	Prospective registry	38	19 (50%)	19 (50%)	73 ± 7	Mean ± SD: 5.0 ± 1.3	Mean ± SD: 4.2 ± 1.0	23 IPH 9 SDH 6 SAH	Median 637 days, minimum 60 days
Nielsen-Kudsk/2017	Retrospective observation	151	99 (65.6%)	52 (34.4%)	71.9 ± 8.7	Mean ± SD: 3.9 ± 1.5	Mean ± SD: 4.2 ± 0.8	Not reported	Median: 203 days; IQR: 99–982
Tzikas/2017	Prospective registry	198	138 (70%)	60 (30%)	73.7 ± 7.9	Mean ± SD: 4.5 ± 1.5	Mean ± SD: 3.5 ± 1.1	Not reported	Not reported
Renou/2017	Prospective observation	46	29 (63%)	17 (37%)	73.7 ± 8.4	Mean ± SD: 5.23 ± 1.12	Mean ± SD: 4.00 ± .95	43 IPH 3 others	Mean ± SD: 7 ± 4 mo
Martínez-Domeño/2017	Retrospective observation	9	7 (77.7%)	2 (22.3)	72.7 ± 8.2	Median 4	Median 3	Not reported	4 with 1 month rest not reported
Cru-Gonzal/2017	Retrospective observation	47	25 (53.1%)	22 (46.9%)	80 ± 6	Mean ± SD: 5 ± 1	Mean ± SD: 4±1	34 IPH 10 SDH 2 SAH 1 microhemorrhages	Less than 3 months 11 More than 3 months 36
Fahmy/2016	Retrospective observation	26	16 (61.5%)	10 (38.5%)	76 ± 7.0	Mean ± SD: 4.9 ± 1.7	Mean ± SD: 4.4 ± 0.6	24 IPH 2 ocular hemorrhage	Mean ± SD: 30 ± 48 mo
Horstman/2014	Prospective observation	20	14 (70%)	6 (30%)	72.6 ± 5.8	Mean: 4.5 ± 1.4	Mean ± SD: 4.7 ± 1.0	15 IPH 4 SDH 1 SAH	Mean ± SD: 23.1 ± 28.6 mo

IPH, intraparenchymal hemorrhage; SAH, subarachnoid hemorrhage; SDH, subdural hematoma.

**Table 2:** Follow-up outcomes.

Author/year	Follow-up duration	Device-related thrombus	Ischemic stroke	Recurrent intracranial hemorrhage	Mortality
Ajmal/2020	27 months	0	0	0	0
Fayos-Vidal/2020	15 months (3–24 months)	0	0	0	0
Pouru/2020	3.6 years median	Not reported	Stroke 8	7	22
TIA 4	7	22	0	0	1
Hucker/2019	6 months	0	0	0	1
Hutt/2019	13.4 months (quartiles 8–19)	1	0	0	0
Nielsen-Kudsk/2017	182 days (25%/75% quartile: 88/372 days)	Not reported	2	1	2
Tzikas/2017	18.4 ± 12.0 months	1.7% (3)	0	1	0
Renou/2017	12 ± 7 months.	1	0	1	0
Martínez-Domeño/2017	15 months (range 3 to 26)	Not reported	0	0	0
Cru-Gonzal/2017	28 months (15–48)	0	1	1	0
Fahmy/2016	11.9 ± 13.3 months	0	1	0	1
Horstman/2014	11.9 ± 13.3 months	1	0	0	0

**Table 3:** Type of devices and antithrombotic regimen after device implant.

Author/year	Type of devices used	Antithrombotic used after device implant	Duration of anticoagulation	Duration of antiplatelet
Ajmal/2020	Watchman	VKA 11 DOACs 5	1.5	DAPT 4.5 mo ASA lifelong

Fayos-vidal/2020	Amplatzer Amulet 7 Amplatzer Cardiac Plug 2	ASA or clopidogrel 5 Not reported 4	N/A	6 months single agent

Pouru/2020	Watchman 2 Amplatzer Amulet 60 Amplatzer Cardiac Plug 42	VKA 1 DOAC 10 LMWH 23 ASA 72el 4 ASA + clopidogrel 19 ASA + dipyridamole 2 ASA + Plavix + dipyridamole 2	≤14 days in 17 pts (50%) ≤1 month in 31 pts (91%)	Clopidogrel ≤ 1 month in 21 pts ASA for ≤6 months in 62 pts After 6 months ASA 28, ASA + dipyridamole 4

Hucker/2019	Watchman	VKA 18 DOAC 32 OAC + ASA 27 (out of 50 on OAC) DAPT 12	1.5	DAPT 4.5 mo ASA lifelong

Hutt/2019	Watchman	VKA 21 (55%) DOAC 17 (45%)	1.5	DAPT for 4.5 months 15 ASA lifelong

Nielsen-Kudsk/2017	Amplatzer Cardiac Plug and Amplatzer Amulet	Clopidogrel 64 (62.1%) ASA 36 (31.1%) None 7 (6.8%)	N/A	6 months

Tzikas/2017	Amplatzer Amulet	DOAC 3, VKA 14 Clopidogrel 10, ASA + LMWH 6, ASA + DOAC 1 ASA + VKA 9, DAPT 22, ASA84, triple therapy 1, LMWH 24, no treatment 24	Not reported	Not reported

Renou/2017	Watchman and Amplatzer Cardiac Plug used; no numbers given	DAPT 1 ASA 43	N/A	DAPT 6 months ASA lifelong

Martínez-Domeño/2017	2 Amplatzer Amulet 7 Amplatzer Cardiac Plug	5 Plavix, 4 aspirin	N/A	Not reported

Cru-Gonzal/2017	Watchman 24, Amplatzer Amulet 21 Amlatzer Cardiac Plug 2	DAPT 38 LMWH 2 Clopidogrel 1 ASA 4	1.5	1.5

Fahmy/2016	Watchman 9 Amlatzer Amulet 5 Amlatzer Cardiac Plug 12	DAPT 24 Clopidogrel 1 ASA 1	N/A	12 DAPT 1 mo 11 DAPT 3 mo 1 DAPT 6 mo

Horstman/2014	Amplatzer Cardiac Plug	VKA 1 DAPT 3 mo	3 mo	DAPT 19 ASA lifelong

VKA: vitamin K antagonist; DOACs: direct oral anticoagulants; ASA: acetylsalicylic acid; LMWH: low molecular weight heparin; DAPT: dual antiplatelet therapy; OAC: oral anticoagulant.

## Data Availability

The data used to support the findings of this study are available in the Supplementary Material file.

## Supplementary Materials

Supplementary Materials
*Supplementary Material 1*. Study protocol. *Supplementary Material 2*. Data. *Supplementary Material 3*. Quality assessment of studies. .
