# Supplementary material for: Feasibility of Left Atrial Appendage Closure in Atrial Fibrillation Patients with a History of Intracranial Bleeding: A Systematic Review of Observational Studies
Source: J Interv Cardiol. 2020 Nov 6;2020:1575839. doi: 10.1155/2020/1575839 (PMC7747006; doi:10.1155/2020/1575839)
Supplement: Supplementary Materials — Supplementary Material 1. Study protocol. Supplementary Material 2. Data. Supplementary Material 3. Quality assessment of studies. . [file 1575839.f1.zip › 1575839.f1/Supplement 1. Study Protocol.docx]

**Title**: ''Feasibility of Left Atrial Appendage Closure in Atrial Fibrillation Patients with History of Intracranial Bleeding: A Systematic Review of Observational Studies''

Types of studies
Case reports, Case series, observational Studies, Registry Data (No randomized trial has been done so far)

**All studies with LAA closure and Previous Intracranial Hemorrhage Will be Included**.

Types of participants
Human subjects with history of Intracranial hemorrhage

Types of interventions
Left Atrial Appendage (LAA) Closure or Watchman/Amplatzer Amulet Device Implants/Amplatzer cardiac plug

Types of outcome measures
The outcomes of interest will be the number of total bleeding events, Intracranial bleeding events and Mortality

Statistical Analysis:

Descriptive statistics, with medians and ranges for continuous variables and frequencies and percentages for dichotomous variables.

**List of Intracranial Hemorrhage**

1. Intracranial Hemorrhage
2. Intraparenchymal hemorrhage
3. Subdural hemorrhage or hematoma
4. Subarachnoid Hemorrhage
5. Extradural Hemorrhage

**List of Intervention:**

1. Left atrial appendage closure

2. Left Atrial appendage occlusion

3. Watchman Device implant

4. Amplatzer Amulet Cardiac Plug Implant
