# Supplementary material for: Feasibility of Left Atrial Appendage Closure in Atrial Fibrillation Patients with a History of Intracranial Bleeding: A Systematic Review of Observational Studies
Source: J Interv Cardiol. 2020 Nov 6;2020:1575839. doi: 10.1155/2020/1575839 (PMC7747006; doi:10.1155/2020/1575839)
Supplement: Supplementary Materials — Supplementary Material 1. Study protocol. Supplementary Material 2. Data. Supplementary Material 3. Quality assessment of studies. . [file 1575839.f1.zip › 1575839.f1/Supplement 2 SearchDocumentation.docx]

**Left Atrial Appendage Closure in Atrial Fibrillation with ICH**

| **Resource Searched** | **Notes** | **Date Searched** | **Number of Results** |
| --- | --- | --- | --- |
| Ovid MEDLINE(R) and Epub Ahead of Print, In-Process & Other Non-Indexed Citations, Daily and Versions(R) 1946 to April 02, 2020 |  | 04/03/2020 | 2,766 |
| Embase.com Embase (1974-Present) |  | 04/03/2020 | 3,636 |
| [Cochrane Central Register of Controlled Trials](https://www-cochranelibrary-com.ezproxy3.library.arizona.edu/)-Issue 4 of 12, April 2020 |  | 04/03/2020 | 211 |
| Web of Science Core Collection- Science Citation Index Expanded (1900-Present) |  | 04/03/2020 | 1,571 |
| Web of Science Core Collection- Conference Proceedings Citation Index (1990-Present) |  | 04/03/2020 | 189 |
| Scopus |  | 04/03/2020 | 1,341 |
| Global Index Medicus |  | 04/03/2020 | 461 |
| ClinicalTrials.gov |  | 04/03/2020 | 204 |
| TOTAL COMBINED |  |  | 10,379 |
| Duplicates Found |  |  | 3,178 |
| TOTAL (dupes removed/available for screening) |  |  | 7,201 |

**Ovid MEDLINE(R) and Epub Ahead of Print, In-Process & Other Non-Indexed Citations, Daily and Versions(R) <1946 to April 02, 2020>**

| **#** | **Searches** | **Results** |
| --- | --- | --- |
| 1 | Atrial Fibrillation/ | 54053 |
| 2 | ((atrial or atrium or auricular) adj (fibrillation or fibrilacion)).tw. | 68544 |
| 3 | 1 or 2 | 79652 |
| 4 | Atrial Appendage/ | 3226 |
| 5 | Septal Occluder Device/ | 2681 |
| 6 | ((appendage or LAA) adj5 (obliteration or closure or occlusion or occluder or occluding or exclusion or device)).tw. | 1892 |
| 7 | LAAO.tw. | 350 |
| 8 | LAAC.tw. | 150 |
| 9 | "occluder device".tw. | 533 |
| 10 | "occlusion device".tw. | 382 |
| 11 | Lariat.tw. | 706 |
| 12 | Watchman*.tw. | 422 |
| 13 | Amplatzer*.tw. | 2817 |
| 14 | Amulet.tw. | 179 |
| 15 | "Cardiac plug".tw. | 169 |
| 16 | LAmbre.tw. | 34 |
| 17 | PLAATO.tw. | 49 |
| 18 | WaveCrest.tw. | 11 |
| 19 | Coherex.tw. | 14 |
| 20 | Ultrasept.tw. | 6 |
| 21 | Occlutech.tw. | 103 |
| 22 | Cardioblate.tw. | 21 |
| 23 | "Sierra Ligation".tw. | 0 |
| 24 | Aegis.tw. | 586 |
| 25 | Atriclip.tw. | 29 |
| 26 | or/4-25 | 9838 |
| 27 | 3 and 26 | 2766 |

**Embase Session Results (3 Apr 2020)**

| No. | Query | Results |
| --- | --- | --- |
| #28 | #27 AND [embase]/lim NOT ([embase]/lim AND [medline]/lim) | **3636** |
| #27 | #3 AND #26 | **6852** |
| #26 | #4 OR #5 OR #6 OR #7 OR #8 OR #9 OR #10 OR #11 OR #12 OR #13 OR #14 OR #15 OR #16 OR #17 OR #18 OR #19 OR #20 OR #21 OR #22OR #23 OR #24 OR #25 | **17187** |
| #25 | atriclip:ti,ab | **68** |
| #24 | aegis:ti,ab | **883** |
| #23 | 'sierra ligation':ti,ab | **0** |
| #22 | cardioblate:ti,ab | **43** |
| #21 | occlutech:ti,ab | **227** |
| #20 | ultrasept:ti,ab | **16** |
| #19 | coherex:ti,ab | **35** |
| #18 | wavecrest:ti,ab | **28** |
| #17 | plaato:ti,ab | **64** |
| #16 | lambre:ti,ab | **55** |
| #15 | 'cardiac plug':ti,ab | **418** |
| #14 | amulet:ti,ab | **343** |
| #13 | amplatzer*:ti,ab | **5052** |
| #12 | watchman*:ti,ab | **1061** |
| #11 | lariat:ti,ab | **893** |
| #10 | 'occlusion device':ti,ab | **625** |
| #9 | 'occluder device':ti,ab | **853** |
| #8 | laac:ti,ab | **386** |
| #7 | laao:ti,ab | **574** |
| #6 | ((appendage OR laa) NEAR/5 (obliteration OR closure OR occlusion OR occluded OR occluding OR exclusion OR device)):ti,ab | **3518** |
| #5 | 'heart atrium appendage'/exp | **8326** |
| #4 | 'left atrial appendage closure device'/exp | **1747** |
| #3 | #1 OR #2 | **168309** |
| #2 | ((atrial OR atrium OR auricular) NEAR/1 (fibrillation OR fibrilacion)):ti,ab | **125071** |
| #1 | 'atrial fibrillation'/exp | **157976** |

**COCHRANE CENTRAL
Search Name: LAAC Afib SR 2020**

**Last Saved: 02/04/2020 11:43:46**

**ID Search**

**#1 MeSH descriptor: [Atrial Fibrillation] this term only**

**#2 ((atrial OR atrium OR auricular) NEAR/1 (fibrillation OR fibrilacion)):ti,ab**

**#3 #1 OR #2**

**#4 MeSH descriptor: [Atrial Appendage] this term only**

**#5 ((appendage OR laa) NEAR/5 (obliteration OR closure OR occlusion OR occluded OR occluding OR exclusion OR device)):ti,ab**

**#6 LAAO:ti,ab**

**#7 LAAC:ti,ab**

**#8 "occluder device":ti,ab**

**#9 "occlusion device":ti,ab**

**#10 Lariat:ti,ab**

**#11 Watchman*:ti,ab**

**#12 Amplatzer*:ti,ab**

**#13 Amulet:ti,ab**

**#14 "Cardiac plug":ti,ab**

**#15 LAmbre:ti,ab**

**#16 PLAATO:ti,ab**

**#17 WaveCrest:ti,ab**

**#18 Coherex:ti,ab**

**#19 Ultrasept:ti,ab**

**#20 Occlutech:ti,ab**

**#21 Cardioblate:ti,ab**

**#22 "Sierra Ligation":ti,ab**

**#23 Aegis:ti,ab**

**#24 Atriclip:ti,ab**

**#25 #4 OR #5 OR #6 OR #7 OR #8 OR #9 OR #10 OR #11 OR #12 OR #13 OR #14 OR #15 OR #16 OR #17 OR #18 OR #19 OR #20 OR #21 OR #22 OR #23 OR #24**

**#26 #3 AND #25**

**Web of Science Core Collection
Science Citation Index Expanded**

| **Set** | **Results** |  |
| --- | --- | --- |
| # 3 | [**1,571**](http://apps.webofknowledge.com.ezproxy4.library.arizona.edu/summary.do?product=WOS&doc=1&qid=9&SID=5C5Nvk4qEjINsIIJE1l&search_mode=CombineSearches&update_back2search_link_param=yes) | #2 AND #1  *Indexes=SCI-EXPANDED Timespan=All years* |
| # 2 | [**16,914**](http://apps.webofknowledge.com.ezproxy4.library.arizona.edu/summary.do?product=WOS&doc=1&qid=7&SID=5C5Nvk4qEjINsIIJE1l&search_mode=GeneralSearch&update_back2search_link_param=yes) | **TOPIC:** ((atriclip OR aegis OR 'sierra ligation' OR cardioblate OR occlutech OR ultrasept OR coherex OR wavecrest OR plaato OR lambre OR 'cardiac plug' OR amulet OR amplatzer* OR watchman* OR lariat OR 'occlusion device' OR 'occluder device' OR laac OR laao OR ((appendage OR laa) NEAR/5 (obliteration OR closure OR occlusion OR occluded OR occluding OR exclusion OR device))))  *Indexes=SCI-EXPANDED Timespan=All years* |
| # 1 | [**87,457**](http://apps.webofknowledge.com.ezproxy4.library.arizona.edu/summary.do?product=WOS&doc=1&qid=4&SID=5C5Nvk4qEjINsIIJE1l&search_mode=GeneralSearch&update_back2search_link_param=yes) | **TOPIC:** ((((atrial OR atrium OR auricular) NEAR/1 (fibrillation OR fibrilacion))))  *Indexes=SCI-EXPANDED Timespan=All years* |

**Conference Proceedings Citation Index-Science**

| **Set** | **Results** | Save History / Create Alert  Open Saved History |
| --- | --- | --- |
| # 3 | [**189**](http://apps.webofknowledge.com.ezproxy4.library.arizona.edu/summary.do?product=WOS&doc=1&qid=22&SID=5C5Nvk4qEjINsIIJE1l&search_mode=AdvancedSearch&update_back2search_link_param=yes) | #2 AND #1  *Indexes=CPCI-S Timespan=All years* |
| # 2 | [**2,304**](http://apps.webofknowledge.com.ezproxy4.library.arizona.edu/summary.do?product=WOS&doc=1&qid=19&SID=5C5Nvk4qEjINsIIJE1l&search_mode=AdvancedSearch&update_back2search_link_param=yes) | TS=((atriclip OR aegis OR 'sierra ligation' OR cardioblate OR occlutech OR ultrasept OR coherex OR wavecrest OR plaato OR lambre OR 'cardiac plug' OR amulet OR amplatzer* OR watchman* OR lariat OR 'occlusion device' OR 'occluder device' OR laac OR laao OR ((appendage OR laa) NEAR/5 (obliteration OR closure OR occlusion OR occluded OR occluding OR exclusion OR device))))  *Indexes=CPCI-S Timespan=All years* |
| # 1 | [**14,293**](http://apps.webofknowledge.com.ezproxy4.library.arizona.edu/summary.do?product=WOS&doc=1&qid=16&SID=5C5Nvk4qEjINsIIJE1l&search_mode=AdvancedSearch&update_back2search_link_param=yes) | TS=((((atrial OR atrium OR auricular) NEAR/1 (fibrillation OR fibrilacion))))  *Indexes=CPCI-S Timespan=All years* |

**Scopus**

**(used Advanced Search settings)**

TITLE-ABS ((atrial OR atrium OR auricular) W/1 (fibrillation OR fibrilacion)) AND TITLE-ABS (atriclip OR aegis OR sierra OR cardioblate OR occlutech OR ultrasept OR coherex OR wavecrest OR plaato OR lambre OR "cardiac plug" OR amulet OR amplatzer* OR watchman* OR lariat OR laac OR laao OR ((appendage OR law) W/5 (obliteration OR closure OR occlusion OR occluded OR occluding OR exclusion OR device)))

**Global Index Medicus**[**http://pesquisa.bvsalud.org/gim/advanced/?lang=en**](http://pesquisa.bvsalud.org/gim/advanced/?lang=en)

**Advanced Search Form**

**Title,abstract, subject:**

("atrial fibrillation" OR “atrium fibrillation” OR "auricular fibrillation" OR "fibrilacion auricular")

**AND**

**Title,abstract,subject:**

(appendage OR LAA OR LAAO OR LAAC OR "occluder device" OR "occlusion device" OR Lariat OR Watchman OR Amplatzer OR Amulet OR "Cardiac plug" OR LAmbre OR PLAATO OR WaveCrest OR Coherex OR Ultrasept OR Occlutech OR Cardioblate OR "Sierra Ligation" OR Aegis OR Atriclip)

Results-461

[WPRIM (Western Pacific) (289)](javascript:%20add_filter('db_wprim');)


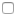


[LILACS (Americas) (89)](javascript:%20add_filter('db_lilacs');)


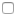


[IMEMR (Eastern Mediterranean) (43)](javascript:%20add_filter('db_imemr');)


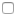


[IMSEAR (South-East Asia) (39)](javascript:%20add_filter('db_imsear');)


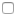


[AIM (Africa) (1)](javascript:%20add_filter('db_aim');)

**ClinicalTrials.gov**

204 Studies found for: **appendage OR LAA OR LAAO OR LAAC OR "occluder device" OR "occlusion device" OR Lariat OR Watchman OR Amplatzer OR Amulet OR "Cardiac plug" OR LAmbre OR PLAATO OR WaveCrest OR Coherex OR Ultrasept OR Occlutech OR Cardioblate OR Aegis OR Atriclip | Atrial Fibrillation**
