# Supplementary material for: Feasibility of Left Atrial Appendage Closure in Atrial Fibrillation Patients with a History of Intracranial Bleeding: A Systematic Review of Observational Studies
Source: J Interv Cardiol. 2020 Nov 6;2020:1575839. doi: 10.1155/2020/1575839 (PMC7747006; doi:10.1155/2020/1575839)
Supplement: Supplementary Materials — Supplementary Material 1. Study protocol. Supplementary Material 2. Data. Supplementary Material 3. Quality assessment of studies. . [file 1575839.f1.zip › 1575839.f1/Supplement 3 NIH Quality Assessment.doc]

| **Criteria** | | **Ajmal/2020** | **Fayos-Vidal/2020** | **Pouru/2020** | **Hucker/2019** | **Hutt/2019** | **Nielsen-** | **Tzikas/2017** | **Renou/2017** | **Martínez-** | **Cru-Gonzal/2017** | **Fahmy, 2016** | **Horstman/** |
| --- | --- | --- | --- | --- | --- | --- | --- | --- | --- | --- | --- | --- | --- |
|  |  |  |  |  |  |  | **Kudsk/2017** |  |  | **Domeño/2017** |  |  | **2014** |
|  | |  |  |  |  |  |  |  |  |  |  |  |  |
| 1. Was the research question or objective in this paper clearly stated? | | Y | Y | Y | Y | Y | Y | Y | Y | Y | Y | Y | Y |
|  |  |  |  |  |  |  |  |  |  |  |  |  |  |
| 2 | Was the study population clearly specified and defined? | Y | Y | Y | Y | Y | Y | Y | Y | Y | Y | Y | Y |
|  |  |  |  |  |  |  |  |  |  |  |  |  |  |
| 3. | Was the participation rate of eligible persons at least 50%? | Y | Y | Y | Y | Y | Y | Y | Y | Y | Y | Y | Y |
|  |  |  |  |  |  |  |  |  |  |  |  |  |  |
|  |  | N | Y | N | Y | Y | Y | Y | Y | Y | Y | Y | Y |
| 4. Were all the subjects selected or recruited from the same or similar populations (including the same | |  |  |  |  |  |  |  |  |  |  |  |  |
| time period)? Were inclusion and exclusion criteria for being in the study prespecified and applied | |  |  |  |  |  |  |  |  |  |  |  |  |
| uniformly to all participants? | |  |  |  |  |  |  |  |  |  |  |  |  |
|  |  |  |  |  |  |  |  |  |  |  |  |  |  |
|  |  | N | N | N | N | N | N | N | N | N | N | N | N |
| **5. Was a sample size justification, power description, or variance and effect estimates provided?** | |  |  |  |  |  |  |  |  |  |  |  |  |
|  |  |  |  |  |  |  |  |  |  |  |  |  |  |
|  |  | Y | Y | Y | Y | Y | Y | Y | Y | Y | Y | Y | Y |
| **6. For the analyses in this paper, were the exposure(s) of interest measured prior to the outcome(s)** | |  |  |  |  |  |  |  |  |  |  |  |  |
| **being measured?** | |  |  |  |  |  |  |  |  |  |  |  |  |
|  |  |  |  |  |  |  |  |  |  |  |  |  |  |
|  |  | Y | Y | Y | Y | Y | Y | Y | Y | Y | Y | Y | Y |
| **7. Was the timeframe sufficient so that one could reasonably expect to see an association between** | |  |  |  |  |  |  |  |  |  |  |  |  |
| **exposure and outcome if it existed?** | |  |  |  |  |  |  |  |  |  |  |  |  |
|  |  |  |  |  |  |  |  |  |  |  |  |  |  |
|  |  | N | N | N | N | N | N | N | N | N | N | N | N |
| **8. For exposures that can vary in amount or level, did the study examine different levels of the** | |  |  |  |  |  |  |  |  |  |  |  |  |
| **exposure as related to the outcome (e.g., categories of exposure, or exposure measured as continuous** | |  |  |  |  |  |  |  |  |  |  |  |  |
| **variable)?** | |  |  |  |  |  |  |  |  |  |  |  |  |
|  |  |  |  |  |  |  |  |  |  |  |  |  |  |
|  |  | Y | Y | Y | Y | Y | Y | Y | Y | Y | Y | Y | Y |
| **9. Were the exposure measures (independent variables) clearly defined, valid, reliable, and** | |  |  |  |  |  |  |  |  |  |  |  |  |
| **implemented consistently across all study participants?** | |  |  |  |  |  |  |  |  |  |  |  |  |
|  |  |  |  |  |  |  |  |  |  |  |  |  |  |
|  |  | N | N | N | N | N | N | N | N | N | N | N | N |
| **10. Was the exposure(s) assessed more than once over time?** | |  |  |  |  |  |  |  |  |  |  |  |  |
|  |  |  |  |  |  |  |  |  |  |  |  |  |  |
|  |  | Y | Y | Y | Y | Y | Y | Y | Y | Y | Y | Y | Y |
| **11. Were the outcome measures (dependent variables) clearly defined, valid, reliable, and** | |  |  |  |  |  |  |  |  |  |  |  |  |
| **implemented consistently across all study participants?** | |  |  |  |  |  |  |  |  |  |  |  |  |
|  |  |  |  |  |  |  |  |  |  |  |  |  |  |
|  |  | N/A | N/A | N/A | N/A | N/A | N/A | N/A | N/A | N/A | N/A | N/A | N/A |
| **12. Were the outcome assessors blinded to the exposure status of participants?** | |  |  |  |  |  |  |  |  |  |  |  |  |
|  |  |  |  |  |  |  |  |  |  |  |  |  |  |
|  |  | Y | Y | Y | Y | Y | Y | Y | Y | Y | Y | Y | Y |
| **13. Was loss to follow-up after baseline 20% or less?** | |  |  |  |  |  |  |  |  |  |  |  |  |
|  |  |  |  |  |  |  |  |  |  |  |  |  |  |
|  |  | N/A | N | N | N | N | Y | N | N | Y | Y | N | Y |
| **14. Were key potential confounding variables measured and adjusted statistically for their impact on** | |  |  |  |  |  |  |  |  |  |  |  |  |
| **the relationship between exposure(s) and outcome(s)?** | |  |  |  |  |  |  |  |  |  |  |  |  |
|  |  |  |  |  |  |  |  |  |  |  |  |  |  |
